# Regular Distribution Inhibits Generic Numerosity Processing

**DOI:** 10.3389/fpsyg.2018.02080

**Published:** 2018-10-31

**Authors:** Wei Liu, Yajun Zhao, Miao Wang, Zhijun Zhang

**Affiliations:** ^1^School of Education, Yunnan Minzu University, Kunming, China; ^2^School of Sociology and Psychology, Southwest University for Nationalities, Chengdu, China; ^3^Department of Psychology and Behavioural Sciences, Zhejiang University, Hangzhou, China

**Keywords:** numerosity perception, element distribution, connectedness effect, individuation, texture specificity

## Abstract

This study investigated the role of pattern regularity in approximate numerical processing. Experiment 1 demonstrated that the change in stimulus size has a distinct effect on the adaptation aftereffect for random and regular patterns. For regular patterns, adapting to large patterns and being tested with small patterns caused stronger aftereffects than the reverse treatment, in which the participants adapted to small patterns and were tested with large patterns. For random patterns, this effect was absent. Experiment 2 revealed a distinct connectedness effect on the numerosity processing of random and regular patterns. For random patterns, reference stimuli were perceived to contain fewer items when the dots were connected by lines than when they were not connected, and the number of items in the connected reference was further underestimated when the participants adapted to unconnected patterns with the same number of dots. For regular patterns, this effect was absent. Distinct mechanisms were thus suggested for the numerosity coding of random and regular patterns. For random patterns, the change in primary texture features would be abstracted from numerosity processing, while connectedness could affect this coding by affecting the processing of numerical unit individuation. For regular patterns, generic numerosity processing is inhibited, and numerical judgments appear to be inferred from the visual processing results of texture features such as dot size or the distance between adjacent dots.

## Introduction

Numerosity cognition is accompanied by the processing of a combination of visual features (Dehaene, 1992; Franconeri et al., 2009). Previous studies have suggested the independence of numerosity processing from the processes associated with texture features, and the abstraction process is suggested to be part of numerosity coding (Burr and Ross, 2008a,b; Liu et al., 2012, 2013). However, these studies have been challenged by other studies indicating that perceived numerosity is affected by some visual features, such as size, contrast, and density (Dakin et al., 2011; Raphael et al., 2013; Raphael and Morgan, 2015). Numerosity adaptation, in which the numerosity of the adaptor affects the observer’s perception of quantity, can be inferred from the change in perceived numerosity before and after adaptation (adaptation aftereffect). Numerosity adaptation is proposed as evidence of an independent numerosity processing mechanism. However, other researchers have argued that this adaptation could occur via more general texture-like mechanisms, relying on features such as dot size or texture density adaptation (Durgin, 2008).

The interaction between visual properties and numerosity coding seems to contradict the idea that numerosity processing occurs through an independent mechanism. Numerosity processing has been proposed to consist of several steps that involve distinct levels (Liu et al., 2013; Zhang et al., 2014). One way to explain the mentioned contradiction is to analyze the interaction at a specific level of numerical processing.

Numerosity processing begins with primary texture analyses. The combined computation results for surrogate features, such as size, density, and the average distance between adjacent dots, are first processed by the visual system. Common bases in processing suggest a pathway between numerosity processing and texture processing, and the interaction may occur mainly at the primary level. In a study by Anobile et al. (2014), participants were asked to compare the number or density of pairs of dot arrays, and the Weber fraction was analyzed. With moderate density, the thresholds increased with numerosity. When the dots became denser, a new pattern of change appeared, suggesting a density-processing mode, regardless of whether participants compared the numerosity or the density of stimulus pairs. As the dots became denser, it became difficult to separate individual dots as numerical units within the crowded texture. Under that condition, numerical cognition was inhibited, and density cognition superseded this processing (Anobile et al., 2014, 2015). When numerical coding is inhibited, stimulus processing may consist of no more than texture processing, which is frequently affected by visual features (Liu et al., 2017).

As visual information is processed from primary to higher levels along the ventral pathway, the presentation of the information transforms from a specific to an abstract format (Dehaene and Changeux, 1993), and the underlying neuronal bases shift from simple to complex (Liu et al., 2017). Generic numerosity processing involves the function of high-level processes such as individuation, abstraction, and numerical unit representation (Liu et al., 2017). The existence of individuation in numerosity processing can be demonstrated by the connectedness effect. When randomly distributed dots are connected by lines, the perceived magnitude is significantly reduced. Two connected dots are considered to be one when observers compare the number of dots (Franconeri et al., 2009; He et al., 2009, 2015; Milne et al., 2013). Adaptation causes a further reduction in the estimated numerosity of connected dots. In a study by Fornaciai et al. (2016), adaptation to a 20-dot pattern (the same number of dots as in the reference) caused a further reduction in the estimated numerosity of the reference, in which two dots were connected as one pair. This fact suggests that adaptation to numerosity acts on perceived numerosity and that magnitude estimation is based on the individuation of items.

The individuation and presentation of numerical units are necessary in numerosity processing (Gallistel and Gelman, 2000). The inhibition of individuation is synchronic with the inhibition of generic numerosity processing (Liu et al., 2013, 2017). A crowding-like effect may inhibit numerosity processing because dots are too dense to be individuated (Anobile et al., 2014). A high degree of regularity in the distribution of dots (e.g., dots spaced at a uniform distance or aligned in rows) could be another way to inhibit numerosity processing because dots in such a distribution are also difficult to individuate. The overall configuration emphasizes meaningful information and observers are likely to understand the pattern by analyzing the spatial relationships between one dot and its fellows in another “neighborhood” instead of by separating a single dot and analyzing it without context (Liu et al., 2017). A distinct adaptation aftereffect was revealed in the numerosity processing of randomly and regularly distributed dots, suggesting this inhibition in the coding of regular dots. The numerosity adaptation aftereffect was immune to change in the orientation of the elements between adaptors and tests and, furthermore, showed binocular transfer (Durgin, 2001; Harris et al., 2011; Sweeny et al., 2011) in the coding of randomly distributed patterns. However, the adaptation aftereffect was specific to the change in the orientation of the elements and exhibited monocular transfer in the coding of regularly distributed patterns (Liu et al., 2017). Numerosity processing should not be generic based on the visual coding of regular patterns.

Texture coding typically interacts at the primary processing level, whereas individuation involves higher levels of activity (Liu et al., 2017). If the distinguishable processes exclusively pertaining to numeral coding, such as individuation or abstraction, are what determine the independence of numerosity cognition, then the arguments claiming that various visual features affect numerosity processing would not necessarily be contradictory. In our 2017 study, it was proposed that element orientation has distinct effects on numerosity processing in random and regular patterns and that compared with random dots, regularly distributed dots inhibit high-level numeral processing. Evidence showing dissociation in numerosity adaptation between the coding of random and regular patterns would convincingly support the case that generic numerosity coding is independent of texture coding and that numerosity coding interacts with texture coding when certain processes are inhibited. In the current study, converging evidence was collected to support the hypothesis that distinct mechanisms control the coding of random and regular patterns. Moreover, we provided further evidence supporting the case that numerosity processing of regular patterns depends on analyses of surrogate features and that perceived numerosity can be inferred from the processing results of certain features of visual arrays, such as dot size or distance. In addition, we collected clearer evidence suggesting that individuation was inhibited in the numerosity processing of regular patterns.

Two experiments were conducted using the adapting paradigm (Burr and Ross, 2008a; Fornaciai et al., 2016). If generic numerosity processing is inhibited when regularly distributed patterns are coded, then it is possible that numerosity estimation is inferred via texture-like mechanisms, such as estimation of the size of the dots or the distance between them (Sophian, 2007). Therefore, the element size relationship between adaptors and test stimuli could affect the numerosity adaptation aftereffect for regularly distributed patterns (Experiment 1), although size immunity has been confirmed in numerosity adaptation for randomly distributed patterns (Burr and Ross, 2008a; Liu et al., 2012). In addition, it has been proposed that individuation is inhibited with regard to the numerosity estimation of regular patterns (Liu et al., 2017). We made further efforts to investigate whether individuation is inhibited with regard to numerosity adaptation. We proposed that when the number of dots is equal in a regularly distributed reference and adaptor, even if the dots in the reference are connected, the adaptors will cause no reduction in the estimated magnitude of the reference (Experiment 2), although such a reduction has been revealed for randomly distributed dots (Fornaciai et al., 2016).

## Experiment 1: the Element Size Specificity of Numerosity Adaptation with Random and Regular Dots

Experiment 1 investigated whether adaptors with elements whose size was different from those in tests would affect the adaptation aftereffect and whether such an effect would vary between adaptation to random and regular patterns. A paradigm similar to that in the previous study (Burr and Ross, 2008a) was adopted to investigate the numerosity adaptation aftereffect.

### Methods

#### Statement

For all experiments, all administered measures and tested experimental conditions were reported. All recorded data from the participants were included in the calculation. Missing data (responses after 1,000 ms in the response window) were excluded from the total set of responses when the selection probability for the point of subjective equality (PSE) was calculated. For each participant, the missing data amounted to less than 3%.

#### Ethics Statement

The data in Experiments 1 and 2 were analyzed anonymously. All adults in this study’s experiments provided their informed consent in both verbal and written forms, and they were compensated for their participation. The ethics committee of Yunnan Minzu University approved this study.

#### Participants

The sample sizes in our previously published study with a similar paradigm (Liu et al., 2017) were taken into consideration. We collected data from 16 participants in each experiment because the abovementioned study showed that this sample size yields ample power. The participants had either normal or corrected-to-normal vision, and they were right-handed. Six males and 10 females (age range = 19–32 years) participated in Experiment 1.

#### Apparatus

The stimuli were displayed using E-Prime 1.0 on a 17″ monitor (Philips, flat-screen) with a resolution of 1,024 × 768 pixels and a refresh rate of 85 Hz. The experiments were conducted in a dark room, and the viewing distance was approximately 55 cm.

#### Stimuli

**FIGURE 1:**
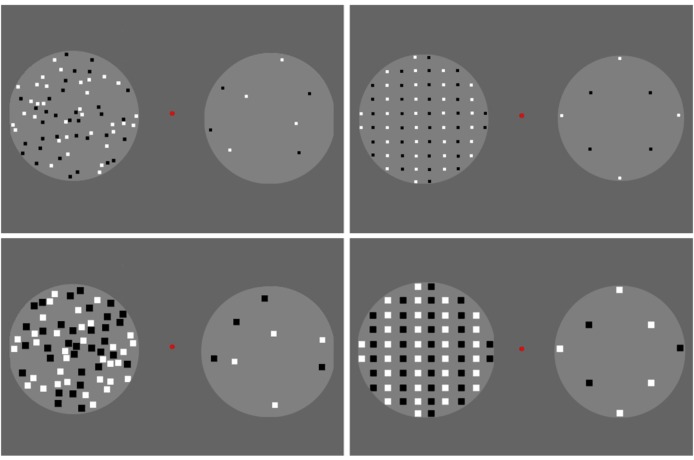
Adaptors used in Experiment 1. The randomly distributed adaptors are shown on the left-hand side, and the regularly distributed (vertical queues) adaptors are shown on the right-hand side. The small adaptors are shown in the upper row, and the large adaptors are shown in the lower row. Dots were generated within two-fixed circles. A total of 68 dots were displayed in one circle, and eight dots were arranged in the other.

Stimuli were generated using Walk Script 1.0 (ZJU Walkinfo Co., Ltd., Hangzhou, China). During the experiment, stimulus patterns were all presented within two-fixed circles in the middle of the computer screen (Figure 1). Each grayscale pattern (RGB: 128, 128, 128) had a diameter of 300 pixels and was presented against a dark-gray (RGB: 120, 120, 120) background. In the adaptation stage, the two circles served to display the adaptors; in the testing stage, they served to display the reference and test stimuli.

For adaptors, there were 68 rectangular dots presented in one circle and 8 in the other (Figure 1). In each circle, half of the dots were white, and the other half were black. The dots in the adaptors were randomly distributed in the “random” condition and were classified into vertical queues by color in the “regular” condition. Each dot in the adaptors was 6 × 6 pixels in the “small” condition and 14 × 14 pixels in the “large” condition. Note that no more than 68 dots were assigned in the adaptors because increasing density would cause increasing difficulty in separating numerical units when more dots were included in the circle, especially, when the “large” adaptors were presented.

Each reference contained 40 dots, which were similar to those in the adaptors. In other words, there were references in which the dots were of small or large size, placed in either random or regular spatial distributions. Within each treatment, the distribution of the dots was kept constant between adaptors and references (random-random or regular-regular), while the dot size differed (small-large or large-small).

For tests, the size and distribution of the dots were kept identical to their references, while the numbers of dots varied. An equidistant logarithmic scale was adopted to decide the numbers of test dots (Dehaene et al., 2008). Moreover, we chose numbers with which a symmetric pattern could be constructed in regular groups; thus, the tests contained 24, 30, 33, 36, 40, 44, 49, 58, or 68 dots. The reference number (40) was assigned in the center of the testing series.

Notably, there was only one distribution pattern for each “regular” stimulus with a certain number of dots (*m* columns and *n* rows). Therefore, we also adopted only one picture for each random stimulus, such that equivalent familiarity could be induced for random and regular conditions when the participants performed the experiment. In total, 4 adaptors, 4 references, and 36 test patterns were generated in Experiment 1.

#### Procedure

We adopted a 2 (dot distribution pattern: random/regular) × 2 (dot size relationship: large-small/small-large) within-subjects design. Therefore, the participants compared the numbers of dots after adaptation across four treatments. Moreover, four unadapted pretests (small-random, small-regular, large-random, and large-regular dots) were conducted as baselines, in which the participants performed the testing procedure directly without any adaptation.

**FIGURE 2:**
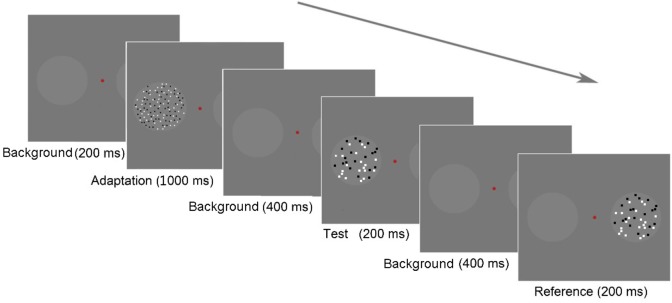
Schematic of the paradigm in Experiment 1. Each trial began with a background frame for 200 ms, followed by an adaptation stage that lasted for 1,000 ms. The test stage began with a background lasting for 400 ms. A test stimulus was displayed in the left circle for 200 ms, followed by a background for 400 ms, and then the reference stimulus was displayed in the right circle for 200 ms. The participants were asked to report, which circle appeared to contain more dots by pressing the appropriate key; if they were uncertain, they were required to guess.

The treatment with adaptation is described in Figure 2 (random, small-large condition). In each treatment, the participants initiated the first trial by pressing the space bar, and a background frame with two circles and a fixation point was visible during the entire procedure. In the adaptation stage, the background frame lasted for 200 ms. Then, the adaptors were presented in the circles for 1,000 ms.

In the testing stage, the background frame was shown for 400 ms at the beginning. Subsequently, a test stimulus was presented in the left circle for 200 ms, followed by the background frame for 400 ms. Then, a reference stimulus was presented in the right circle for 200 ms. Once the reference appeared, the participants should respond to a forced-choice question: “Which circle contained more dots?” They pressed either the “f” key on the keyboard with their left hand, indicating that the left circle contained more dots, or “j” with their right hand, indicating that the right circle contained more dots. In other words, we used a two-alternative forced-choice (2AFC) task to assess numerosity perception. The next trial began either after the participant’s response or after 1,200 ms without a response.

At the beginning of Experiment 1, brief practice trials with feedback were conducted to improve the participants’ familiarity with the formal experiment. Then, the participants completed four pretests in a random sequence before performing the adaptation tasks to create baselines. In the pretest, no adaptors were presented, and each of the 72 trials proceeded directly to the testing stage. After these tasks, the participants began the formal experiment with four treatments, each with 72 trials. The adaptors, reference, and test positions were counterbalanced across participants and were kept identical within treatments for each participant. The sequences of treatments with adaptors were also counterbalanced across participants. Sufficient rest was provided between treatments to avoid fatigue.

### Results

**FIGURE 3:**
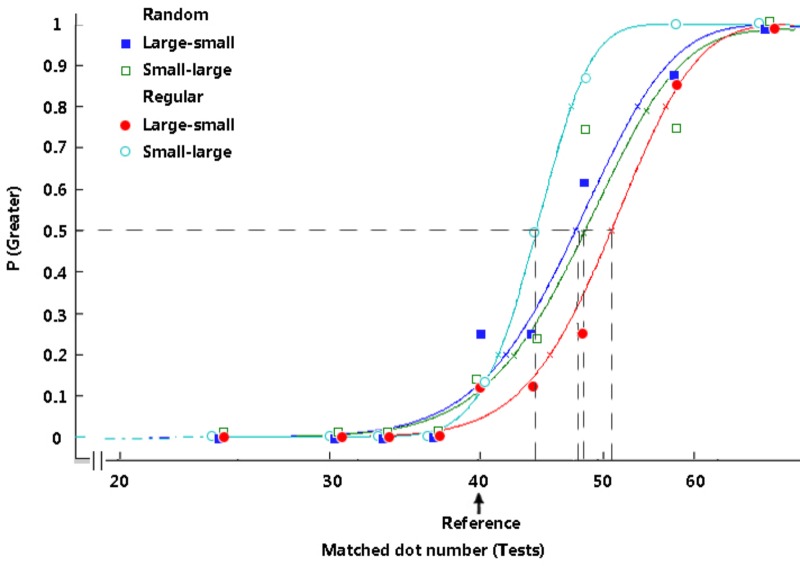
Typical psychometric functions under distinct conditions in Experiment 1. The proportion of trials in which the test stimuli appeared to be more numerous is plotted as a function of the number of test dots, and the vertical dashed lines reveal the PSEs. The arrow indicates the reference number. The participants’ typical responding curves are displayed to indicate the average PSE results. In the random group, filled rectangles, dark-blue curve = large adaptors and small tests; open rectangles, green curve = small adaptors and large tests. In the regular group, filled circles, red curve = large adaptors and small tests; open circles, light-blue curve = small adaptors and large tests.

Cumulative normal models were fitted to the psychometric functions of each participant using the psignifit toolbox version 2.5.41 for MATLAB^1^. The maximum likelihood method (Wichmann and Hill, 2001) was adopted to measure the magnitude of the connectedness effect. The values of the test stimuli (*X*-axis) corresponding to the 50% points were calculated from the fitted curves (Figure 3). These values were the PSEs representing the number of test dots that appeared to be equal to the number of reference dots according to each participant. The change in numerosity perception in the tests is represented by the difference in the PSEs under different circumstances (Table 1). Therefore, the magnitude of the numerosity adaptation aftereffect is revealed by the PSEs under the adaptation conditions minus the PSEs in the pretests.

**Table 1:** The means and SDs for the PSEs in the pretest and adaptation conditions in Experiment 1.

	Test pattern: random				Test pattern: regular			
	Pretest (small)	Pretest (large)	Large–small	Small–large	Pretest (small)	Pretest (large)	Large–small	Small–large
PSE	41.97	42.64	48.03	48.89	41.58	42.40	51.01	44.52
SD	4.03	6.98	7.83	9.81	5.64	4.35	7.59	7.17


*PSE, point of subjective equality; SD represents the standard deviation of the PSE. “Large–small” refers to the treatment in which the participants were exposed to large adaptors and small tests. The rest can be performed in the same manner.*

No significant main effect or interaction was observed between the four pretests. There was a significant difference between the treatment and its baseline (pretest) in the random group for the “large-small” condition, *t*(15) = 3.09, *p* = 0.008, *d* = 0.77, and for the “small-large” condition, *t*(15) = 3.48, *p* = 0.003, *d* = 0.87, as well as a significant difference in the regular group for the “large-small” condition, *t*(15) = 6.69, *p* < 0.001, *d* = 1.67, showing that both the randomly and regularly distributed adaptors affected the participants’ numerosity perception. In most cases, when the presented scene was shifted from adaptors to tests, the number of dots in the circle decreased from 68 to less than 68 (according to the test dot number), decreasing the numbers perceived by the participants in the tests (Burr and Ross, 2008a). Subsequently, the apparent number of dots in the reference (PSE) was overestimated. No significant difference was evident between the treatment and its pretest in the regular group for the “small-large” condition (*p* = 0.151).

**FIGURE 4:**
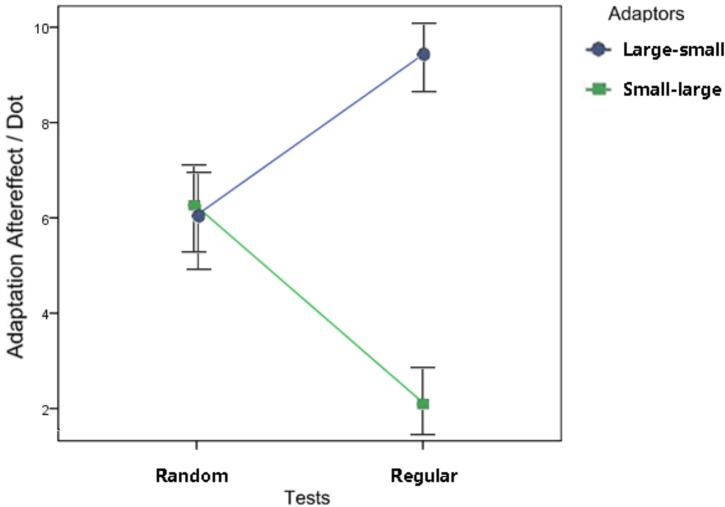
Results of the ANOVA in Experiment 1. A significant interaction was found between the dot distribution (the two shapes on the left of Figure 4 = random adaptors, references, and tests; the other two shapes on the right = regular adaptors, references, and tests) and the size relationship (circles = adapting to large dots and tested by small dots; rectangles = adapting to small dots, and tested by large dots). In the regular groups, a greater adaptation effect was revealed in the large-small condition than in the small-large condition. In the random groups, however, the difference between conditions with those two size relationships was not significant. Error bars represent 1 standard error of the mean.

The adaptation aftereffect was calculated by subtracting the PSEs of treatments from those of their pretests (Liu et al., 2012, 2017). A 2 × 2 repeated-measures ANOVA was conducted with the test patterns (random or regular) and the stimulus size relationship (large-small or small-large) as the independent variables and the adaptation aftereffect as the dependent variable. No significant main effect of the test pattern was found (*p* = 0.810); however, the main effect of the stimulus size relationship, *F*(1, 15) = 14.38, *p* = 0.002, η_p_^2^ = 0.49, and the interaction between the two factors (Figure 4), *F*(1, 15) = 9.93, *p =* 0.007, η_p_^2^ = 0.40, were significant. With regular adaptors and tests, a greater effect was found in the participants’ numerosity perception when they adapted to the large dots and were tested using small dots than when they adapted to small dots and were tested using large dots, *p* < 0.001. When the participants adapted to random dots and their perception was tested using random dots, the size relationship between the adapting and testing stimuli caused no significant difference between treatments, *p* = 0.908.

## Experiment 2: the Effect of Connectedness on Numerosity Adaptation with Random and Regular Dots

Experiment 2 examined the effects of the connectedness of elements on numerosity adaptation, in which the adapting and testing stages were conducted with randomly and regularly distributed dots, respectively. The aftereffect of numerosity adaptation in connected random dots was tested by Fornaciai et al. (2016). A similar paradigm was used in Experiment 2.

### Methods

#### Participants

Six males and 10 females (age range = 20–32 years) participated in Experiment 2.

#### Stimuli

**FIGURE 5:**
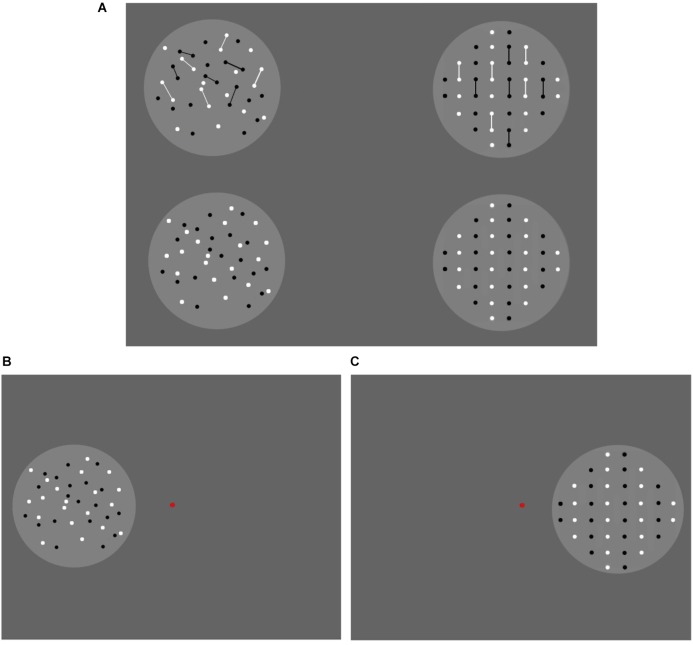
The stimuli used in Experiment 2. The reference patterns are shown in **(A)**. The connected adaptors are displayed in the upper row, and the unconnected adaptors are presented in the lower row. In each row, from left to right, the references in the random and regular groups, respectively. The test patterns were similar to the reference in the lower row, except for the number of dots. The adaptor patterns are shown in **(B)** (the random group) and **(C)** (the regular group). In the adaptation stage, the 40-dot adaptor was presented on one side of the screen. The other side was blank.

Both the reference and the test patterns were arranged within two-fixed circles similar to those in Experiment 1. Four reference patterns were first created, each containing 40 circular dots with a diameter of 12 pixels (Figure 5). In two of the patterns, no lines were included. In the other two patterns, each pattern contained 10 two-pixel-wide line segments of varying length (30–50 pixels). The dots were at least 10 pixels apart. The lines did not cross each other. In Reference 1 (random, connected), the dots were randomly distributed. In each pattern, each individual line linked two adjacent dots to form a connected object (10 lines connected to 20 dots overall). In Reference 2 (random unconnected), the dots were randomly distributed, and no lines were included. In Reference 3 (regular, connected), the dots were arranged into vertical queues. Ten vertical lines were arranged to connect adjacent dots. In Reference 4 (regular, unconnected), the dot presentation was similar to that in Reference 3, and no lines were included. The lines in each random pattern had a varying length, with an average value of 42 pixels, and the lines in each regular pattern had a fixed length of 44 pixels. The connected reference was used for the treatment conditions, and the unconnected reference was used for the baseline conditions. In each condition, the dot distribution in the test patterns was similar to that in the reference patterns (random or regular). No lines were included in the test patterns. The tests contained 18, 24, 30, 33, 36, 40, 44, 49, 58, 68, or 78 dots. Overall, 22 test patterns were generated.

For adaptors, the 40-dot test patterns were adopted. In the random group, 40 dots were randomly distributed in the presentation circle. In the regular group, 40 dots were regularly distributed.

#### Procedure

**FIGURE 6:**
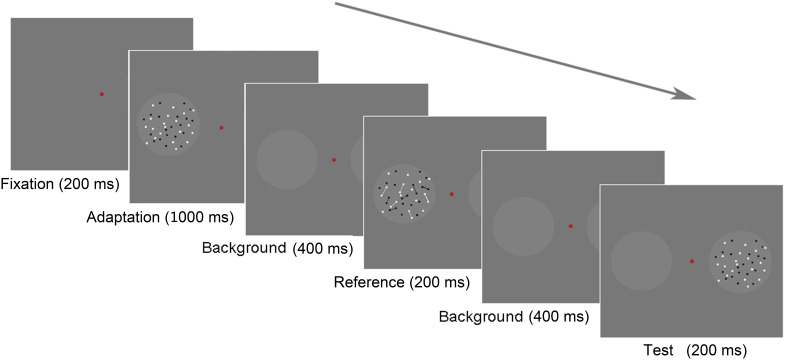
Experiment 2 paradigm. Each trial began with an adaptation stage of 1,000 ms. When the test stage began, there was a background frame lasting for 400 ms. Then, a reference stimulus was displayed in one circle for 200 ms, followed by a test stimulus displayed in the other circle for 200 ms. The two stimuli were separated by a background frame for 400 ms.

We adopted a 2 (stimulus pattern: randomly/regularly distributed elements) × 2 (reference pattern: connected/unconnected dots) within-subjects design. The procedure is described in Figure 6. In general, the procedure was similar to that in Experiment 1. There is one observable difference in the testing stage. In this stage, the reference was presented first in the same position where the adaptor was presented before, followed by the test displayed in the opposite position. The participants were asked to compare the number of dots in the reference and test stimuli, that is, to report which circle contained more dots by pressing “f” or “j,” and they were instructed to ignore the lines (if any) when they were estimating the number of dots.

### Results

**FIGURE 7:**
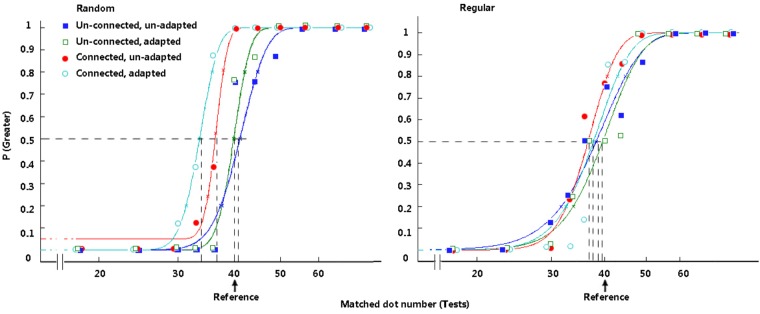
Typical psychometric functions in Experiment 2. The functions in the random group are presented on the left. The functions in the regular group are presented on the right. In each group, filled rectangles, dark-blue curve = treatments with an unconnected reference and without an adaptor; open rectangles, green curve = treatments with an unconnected reference and with an adaptor; filled circles, red curve = treatments with a connected reference and without an adaptor; open circles, light-blue curve = treatments with a connected reference and with an adaptor.

Figure 7 and Table 2 demonstrate the difference in the average PSEs under different circumstances. The magnitude of the connectedness effect is indicated by the baseline PSEs minus the treatment PSEs for each group.

**Table 2:** The means and SDs for the PSEs in each treatment in Experiment 2.

Group	Random				Regular			
Condition	Unconnected Unadapted	Unconnected Adapted	Connected Unadapted	Connected Adapted	Unconnected Unadapted	Unconnected Adapted	Connected Unadapted	Connected Adapted
PSE	40.92	40.00	36.28	34.36	39.47	39.62	37.62	38.53
SD	4.33	3.97	4.63	4.32	3.99	3.92	5.58	4.66


*Unconnected/Connected: treatments in which the reference dots were unconnected/connected by lines. Unadapted/Adapted: treatments without/with the adaptation stage.*

There was no significant difference between the PSE of the baselines (the unconnected and unadapted conditions in the random and regular groups) and the standard value (40), *p* > 0.05. In the random group, when the dots were not connected by lines, no significant PSE difference was revealed between the conditions with and without adaptation (*p* = 0.247). Adapting to an adaptor with an equal reference number did not affect the participants’ numerosity perception of the reference. Compared with the results of the unconnected baseline, a significant difference was revealed when dots were connected by lines, *t*(15) = 4.087, *p* = 0.001, *d* = 1.02. Connection significantly decreased the perceived magnitude for random dots. Importantly, when the participants were comparing the number of dots connected by lines after adaptation, there was a further decrease for PSEs compared with the connected condition without adaptation, *t*(15) = 2.585, *p* = 0.021, *d* = 0.65. This difference indicates that adaptation affected perceived numerosity when the dots were connected in the reference, even though the dot number was equal in the adaptor and the reference. When the presented scene was shifted from the adaptor to the reference, connectedness decreased the perceived magnitude of the reference, and adaptation intensified the reduction in PSE. These results are in accord with previous research results (Fornaciai et al., 2016).

In the regular group, the situation seemed to be different. When dots were not connected, no significant PSE difference was found between circumstances with and without adaptation (*p* = 0.829). When dots were connected, the PSE difference between conditions with and without adaptation was not significant, either (*p* = 0.312).

A marginally significant decrease was found when the reference dots were connected (in the condition without adaptation) compared with the unconnected and unadapted baseline, *t*(15) = 1.889, *p* = 0.078, *d* = 0.47. Here, we provide a discussion of this marginal effect. When we compared the perceived numerosity of the treatments in which the lines were not controlled to be constant, the connectedness effect and/or the appearance of lines could be potential causes for the change in perceived numerosity. In our previous studies, in which the numbers rather than the distribution of lines were counterbalanced in the tests and the reference, the magnitude of the connectedness effect was directly related to the number of connected dot pairs in the random group (8 connected pairs, an 8-dot decrease in PSE), whereas the connection caused only a one- or two-dot decrease in PSEs in the three regular groups (Liu et al., 2017). The distinct magnitude of the decrease effect indicates that connection affects number perception in the random groups by changing numeral unit individuation; in contrast, number perception was affected because lines and connections caused a texture difference between the reference and the tests in the regular groups. In the current study, the decrease caused by lines is one or two dots in the regular group and approximately four dots in the random group. To some extent, the reduction still differs in the two groups, suggesting that the connection effect in the regular group acts differently from that in the random group. Nevertheless, the coding immunity of regular patterns regarding connectedness is mainly supported by that there was no significant difference between the connected treatments with and without adaptation.

## Discussion

The independence of numerosity processing from the processes associated with texture features, such as element size, orientation, and texture, has been confirmed repeatedly by previous studies (Burr and Ross, 2008a,b; Liu et al., 2012, 2013, 2017). This independence demonstrates the involvement of abstraction processing in numerosity coding. In the current study, numerosity adaptation was shown to be independent of the change in element size in Experiment 1. This result is in accord with those of previous studies (Burr and Ross, 2008a; Burr, 2013).

In contrast, Experiment 1 demonstrated that the change in the element size relationship between adaptors and tests could affect numerosity adaptation for regular patterns. Adapting to large patterns and being tested with small patterns (the large-small condition) caused stronger aftereffects than adapting to small patterns and being tested with large patterns (the small-large condition). It is suggested that open space, which refers to the space that is not occupied by elements in a scene, is relevant to numeral comparison. The participants might have referred to non-numerical cues such as open space when they were asked to compare the numerosity of two sets of dots (Sophian, 2007). In Experiment 1, with an equal dot number, open space was inversely proportional to dot size. When the presented scene was shifted from the adaptor to the tests, the open space increased more dramatically under the large-small condition, in which the dot number decreased from 68 to less than 68 (in most cases) and the dot size transferred from 14 × 14 to 6 × 6 (pixel), than under the small-large condition, in which the dot number changed equally but the dot size changed inversely. Comparably, the adaptation aftereffect was revealed to be stronger under the large-small condition. We suggest that, for regular patterns, numerosity adaptation occurs via the adaptation of open space or open distance between adjacent dots. For regular patterns, numerosity estimation may use distance estimation as a reference. Abstraction seems to be inhibited, and texture specificity has been revealed repeatedly (orientation specificity, Liu et al., 2017; size specificity, the current study) in the numerosity coding of regular patterns.

Our previous studies suggested that regularity inhibited generic numerosity processing by inhibiting high-level processing, such as individuation. Experiment 2 provides new evidence for this suggestion. For random patterns, a reduction in magnitude perception was found when dots were connected, and a further reduction was revealed when the participants were asked to perceive the magnitude of the connected reference after adaptation to an adaptor whose dots were equal in number to those of the reference and were not connected. These results, which suggest that numerosity coding and adaptation directly affect perceptual mechanisms sensitive to number, are comparable to those of previous studies (Liu et al., 2012; Fornaciai et al., 2016). For regular patterns, however, the connectedness effect was absent in numerosity adaptation. This absence suggests an inhibition of individuation, which should be located in a higher step of numerosity processing and should be based on the activity of a set of complex neurons (Liu et al., 2017). Compared with the paradigm used in our 2017 study, the adaptation paradigm in the current study provides improved evidence for the absence of a connectedness effect in the coding of regular patterns. Because the appearance of lines was kept constant in the treatments with and without adaptation, texture differences did not disrupt the comparison of perceived numerosity between these treatments.

Generic numerosity processing is likely to involve the activity of abstraction and individuation. When numerosity processing goes from a low to a high level, the primary coding of visual features could be discarded to form an abstract representation of the numerical units (Stoianov and Zorzi, 2011). Additionally, the magnitude estimation is likely based on the distinct number of items that have been individuated (Gallistel and Gelman, 2000). When high-level processing is inhibited by the visual properties of texture, numerosity processing may be indistinguishable from texture processing (Anobile et al., 2014; Liu et al., 2017). It is possible that regular distribution could cause a general inhibition for high-level processing in numerosity coding, including individuation and abstraction. The inhibition must function automatically rather than strategically, as no strategy was encouraged when the participants were asked to passively watch the screen, and they were informed that the adaptors were irrelevant to the tasks in our studies.

There might be a good reason for the inhibition of high-level numerosity processing in regularly distributed patterns. In natural scenes, it is more efficient to inhibit unnecessary (high-level) processing that achieves generic numerosity cognition when we observe regular patterns because it is more likely that we can obtain useful information by classifying “what” than by estimating “how many” (Liu et al., 2017).

More evidence suggesting that numerosity processing and texture processing share a common origin and arrive at distinct destinations could be gathered, for example, by comparing the event-related potential (ERP) component of numerosity and texture coding. Regardless, there will not necessarily be any contradiction in showing that various statistics of the image affect the approximation of numerosity. It is the distinguishable processing pertaining exclusively to numerosity coding, such as abstraction, individuation, unit representation, and spatially associated representation (Fischer, 2003; Liu et al., 2015), that determines the independent mechanism of numerosity cognition.

The inhibition caused by regular patterns, which was revealed repeatedly in our current and previous studies (Liu et al., 2017), suggests an important role of random distribution in generic numerosity processing. Recently, a handful of studies have investigated common factors underlying approximate number system (ANS) acuity and mathematical achievement (Halberda et al., 2008; Chen and Li, 2014). The accurate measure of ANS acuity is important for this line of investigation. The current study provides additional suggestions on the design of tasks that measure the acuity of ANS. To measure the ANS acuity in an accurate manner, it is necessary to adopt a random dot pattern, as regularity in pattern distribution would inhibit generic numerosity coding. Similarly, it is also necessary to adopt a pattern with moderate density, as numerosity coding could also be inhibited by a cloudy-like effect (Anobile et al., 2014).

## Conclusion

Dot size has a distinct effect on numerosity adaptation with random and regular distributed patterns. For random patterns, the change in stimulus size has no effect on adaptation. For regular patterns, adapting to large patterns and being tested with small patterns causes stronger aftereffects than adapting to small patterns and being tested with large patterns. The connectedness effect is different in the adaptation of random and regular patterns. For random patterns, references were perceived to be less numerous when the dots were connected via lines than when they were not connected, and there was a further underestimation of the connected references when the participants adapted to unconnected patterns with the same number of dots. This connectedness effect was absent in the numerosity estimation and the adaptation of regular patterns.

## Author Contributions

WL wrote the article and designed the experiments. YZ and ZZ edited the manuscript. MW collected the data.

## Conflict of Interest Statement

The authors declare that the research was conducted in the absence of any commercial or financial relationships that could be construed as a potential conflict of interest.

## Footnotes

1 http://www.bootstrap-software.com/psignifit/
